# A Novel Compound Targeting Protease Receptor 1 Activators for the Treatment of Glioblastoma

**DOI:** 10.3389/fneur.2018.01087

**Published:** 2018-12-17

**Authors:** Efrat Shavit-Stein, Ehud Sheinberg, Valery Golderman, Shirley Sharabi, Anton Wohl, Shany Guly Gofrit, Zion Zivli, Natalia Shelestovich, David Last, David Guez, Dianne Daniels, Orna Gera, Kate Feingold, Zeev Itsekson-Hayosh, Nurit Rosenberg, Ilia Tamarin, Amir Dori, Nicola Maggio, Yael Mardor, Joab Chapman, Sagi Harnof

**Affiliations:** ^1^Department of Neurology and Joseph Sagol Neuroscience Center, Sheba Medical Center, Tel Hashomer, Sackler Faculty of Medicine, Tel Aviv University, Ramat Gan, Israel; ^2^Department of Neurosurgery, Rabin Medical Center, Sackler Faculty of Medicine, Tel Aviv University, Ramat Gan, Israel; ^3^The Advanced Technology Center, Sheba Medical Center, Tel Hashomer, Ramat Gan, Israel; ^4^Department of Neurosurgery, Sheba Medical Center, Tel Hashomer, Sackler Faculty of Medicine, Tel Aviv University, Ramat Gan, Israel; ^5^Pathology Institute, Sheba Medical Center, Tel Hashomer, Ramat Gan, Israel; ^6^Institute of Thrombosis and Heamostasis, Coagulation Laboratory, Sheba Medical Center, Tel Hashomer, Ramat Gan, Israel; ^7^The Advanced Technology Center, Sheba Medical Center, Tel Hashomer, Sackler Faculty of Medicine, Tel Aviv University, Ramat Gan, Israel; ^8^Robert and Martha Harden Chair in Mental and Neurological Diseases, Sackler Faculty of Medicine, Tel Aviv University, Ramat Gan, Israel

**Keywords:** glioblastoma, thrombin receptor, PAR1, tumor size, edema, survival

## Abstract

Data from human biopsies, *in-vitro* and *in-vivo* models, strongly supports the role of thrombin, and its protease-activated receptor (PAR1) in the pathology and progression of glioblastoma (GBM), a high-grade glial tumor. Activation of PAR1 by thrombin stimulates vasogenic edema, tumor adhesion and tumor growth. We here present a novel six amino acid chloromethyl-ketone compound (SIXAC) which specifically inhibits PAR1 proteolytic activation and counteracts the over-activation of PAR1 by tumor generated thrombin. SIXAC effects were demonstrated *in-vitro* utilizing 3 cell-lines, including the highly malignant CNS-1 cell-line which was also used as a model for GBM *in-vivo*. The *in-vitro* effects of SIXAC on proliferation rate, invasion and thrombin activity were measured by XTT, wound healing, colony formation and fluorescent assays, respectively. The effect of SIXAC on GBM *in-vivo* was assessed by measuring tumor and edema size as quantified by MRI imaging, by survival follow-up and brain histopathology. SIXAC was found *in-vitro* to inhibit thrombin-activity generated by CNS-1 cells (IC_50_ = 5 × 10^−11^M) and significantly decrease proliferation rate (*p* < 0.03) invasion (*p* = 0.02) and colony formation (*p* = 0.03) of these cells. In the CNS-1 GBM rat animal model SIXAC was found to reduce edema volume ratio (8.8 ± 1.9 vs. 4.9 ± 1, *p* < 0.04) and increase median survival (16 vs. 18.5 days, *p* < 0.02 by Log rank Mental-Cox test). These results strengthen the important role of thrombin/PAR1 pathway in glioblastoma progression and suggest SIXAC as a novel therapeutic tool for this fatal disease.

## Introduction

Glioblastoma (GBM) is high-grade glial tumor, and is the most common, aggressive, rapidly progressing malignant primary brain tumor in humans. Extensive surgical approaches in combination with radiation and chemotherapy rarely achieve survival beyond 2 years (1). Many factors contribute to the high mortality rate in patients suffering from these rapidly growing tumors, among them, brain invasion, inflammation, and tumor associated brain edema. Over the years, tremendous advances have been made in the understanding of GBM pathophysiology (2). Nevertheless, no significant changes in survival rates were noted (1, 3).

Thrombin, a well-studied coagulation factor, stimulates vasogenic edema, tumor adhesion and tumor growth in glioma cell lines and in *in-vivo* models (4–12). The action of thrombin in this context is mediated by direct cell activation via a unique receptor belonging to the family of G protein coupled protease activated receptors (PARs), known as PAR1 (13–15). Thrombin, via PAR1 activation, is known to act as a growth factor and proliferation inducer (16, 17).

Numerous findings support the involvement of the thrombin-PAR1 pathway in glioma pathology (8, 13, 18). We have previously found a positive correlation between edema size and PAR1 expression in an animal model of GBM (19). Previous studies have demonstrated that glioma cells express functional thrombin receptor PAR1 (20–23) and produce endogenous thrombin (19). In addition to the direct thrombin-PAR1 effects on glioma cells, PARs play a crucial role in the control of angiogenesis and vascular permeability by regulating the release of endostatin and vascular endothelial growth factor from platelets, thereby affecting tumor growth, inflammation and edema (24–29). In contrast to normal brain, the vasculature of GBM tumors is typically highly permeable with the potential of prothrombin entry from blood into tumor. This exogenous thrombin combines with endogenous thrombin which is known to be physiologically synthesized in the brain and induced by pathologies such as ischemia (30). The thrombin/PAR1 pathway is essential for glioma cell proliferation and survival since PAR1 and thrombin inhibition induce decreased proliferation and increase in cell death (31, 32). There are known limitations for direct PAR1 antagonist use. Blocking PAR1 may lead to both brain dysfunction and increased risk of bleeding (33) as was found for Vorapaxar (34), the use of which was limited due to bleeding side-effects.

We have designed a number of compounds that are based on the thrombin binding-site sequence of PAR1. The experiments described in this study were conducted utilizing a compound which holds the highest potency inhibiting effect on glioma-secreted thrombin and was named SIXAC.

This work describes the *in-vitro* effects of SIXAC on thrombin inhibition, cell proliferation, and invasion. *In-vivo* experiments describe the effects of SIXAC in the CNS-1 rat glioma model. Better understanding of the complex interaction between glioma cells and their immune microenvironment led us to shift away from xenograft tumor models toward models that result in a less immunogenic response such as CNS-1 (35) and are used in an immunocompetent animal. The CNS-1 cell line uniquely shares several key features with human GBM (25). Currently very few publications have exhibited a therapeutic effect while using *in-vivo* orthotopic models, most likely due to these having features similar to human GBM such as diffusely infiltrative and invasive pattern of growth *in-vivo*.

Tumor and edema volume progression as well as survival rate were the main parameters that were studied in this work. Our results indicate beneficial outcome for SIXAC treatment regarding tumor size, surrounding edema and survival in an *in-vivo* orthotopic glioma xenotransplant model in an immunocompetent rodent host.

## Materials and Methods

### TCGA Dataset

The F2R (PAR1) expression data of 542 GBM patients and 10 controls were collected and analyzed by Oncomine software (www.oncomine.org, 10,2018, Thermo Fisher Scientific, Ann Arbor, MI) based on datasets from The Cancer Genome Atlas-Cancer Genome database (TCGA database, https://cancergenome.nih.gov/) (2).

### Molecule Design

**Figure 1:**
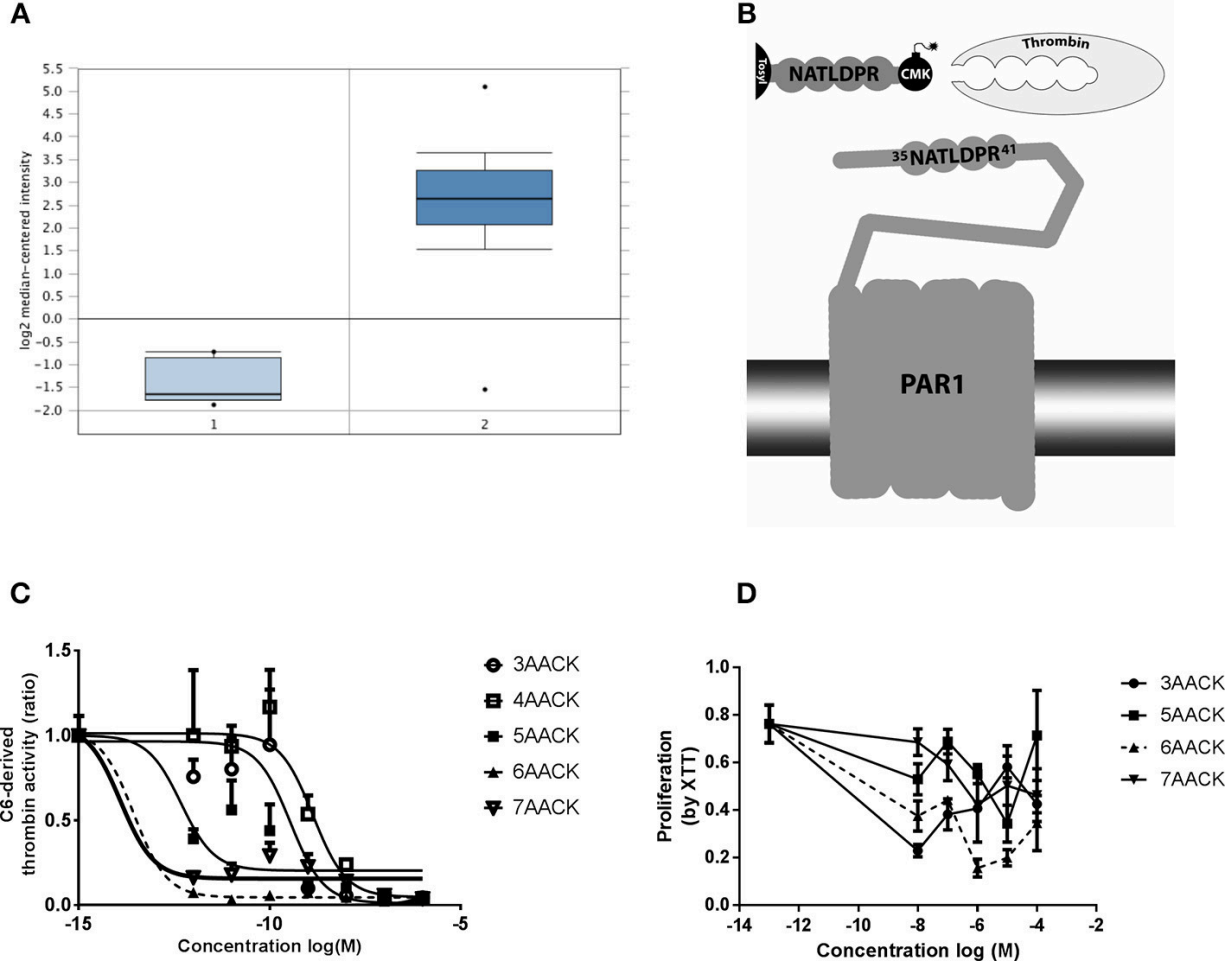
Characterization of novel thrombin inhibitor compounds. **(A)** Expression of F2R gene in GBM patients brains compared to healthy individuals. **(B)** Schematic illustration of the thrombin recognition amino-acid sequence of PAR1 as the logic behind design of molecules that ireversibly interact with thrombin active site by CMK. **(C)** Concentration dependent inhibition of thrombin activity, secreted from C6 glioma cells, by the tested compounds. Nonlinear fit curves are presented. **(D)** Dose-response curves of U87 glioma cells proliferation inhibition rate by the tested compounds. 6AACK (SIXAC) is represented by dashed line. Results are presented as mean ±/+ SEM.

Five molecules were synthesized based on the thrombin recognition site sequence on PAR1 N-terminal side (^35^NATLDPR^41^). The molecules were blocked by a Tosyl group at the N-terminal and a serine active site blocker, chloro-methyl-keton (CMK), as a C-terminal modification (Figure 1B). The five molecules differed regarding the number of amino acids constituting the peptide backbone, between three to seven amino acids, and termed accordingly (3AACK, 4AACK, 5AACK, 6AACK, and 7AACK). The molecules were synthesized and purchased from American Peptide Company (CA, USA) in purity grade of over 96% which was verified by HPLC. Following *in-vitro* characterizations, 6AACK was chosen as the leading molecule and named SIXAC.

### Cell Culture

CNS-1, C6, and U87 rat and human glioma cells were grown in Dulbecco's modified Eagle's medium (DMEM; Bet Haemek, Biological Industries, Israel) supplemented with 10% fetal bovine serum (Bet Haemek, Biological Industries, Israel) and 1% penicillin and streptomycin in a 5% CO2-humidified atmosphere.

### Thrombin Activity Measurements

Thrombin activity was assessed using a fluorogenic thrombin substrate (Bachem I-1560, excitation 360 nm; emission 465 nm). As previously described (19, 36), the reactions were carried out in a black 96-well microplate. All reactions were performed in Tris buffer (in mM: 150 NaCl, 1 CaCl_2_, 50 Tris-HCl: pH 8.0) and in the presence of endopeptidase inhibitors (0.1 mg/ml, bestatin hydrochloride—B8385, Sigma; 200 μM prolylendopeptidase inhibitor II, 537011, Merck Millipore) to eliminate the effect of widely abundant CNS endopeptidases on the assay. Known bovine thrombin concentrations (T-4648, Sigma) were used to create a calibration curve for each experiment. IC50 for inhibition of C6 glioma-generated thrombin activity by the molecules was measured in the medium secreted from the cells as described before (19). Briefly, C6 cells were seeded (5 × 10^4^ cells/ml, 200 μl per well) and allowed to attach the well bottom for 24 h. Then the medium was replaced by FCS-free medium for another 24 h. Medium was transferred to an empty well and the molecules were added at increasing concentrations (10^−15^-10^−6^M). The substrate was added and fluorescence was measured. After deciding on SIXAC as the leading molecule, the IC50 for inhibition was examined in CNS-1 cells in a similar way (10^−12^-10^−5^ M final SIXAC concentrations). Inhibition of commercial thrombin by SIXAC was measured by applying increasing concentrations of SIXAC (10^−12^-10^−5^M final concentration) to wells containing 0.05U/ml thrombin (final concentration). SIXAC stability was tested by sampling SIXAC (400 μM) that was incubated at 37°C at several points in time. The sampled SIXAC was added to a constant concentration of thrombin (0.05 U/ml) and thrombin activity was measured. The total activity was defined as the activity of thrombin without any tested sample. All other measures were calculated as a portion of the total activity.

### Cell Proliferation

Cell proliferation was evaluated using a 2.3-Bis-(2-methoxy-4-nitro-5-sulfophenyl)-2H-tetrazolium-5-carboxyanilide salt (XTT)-based cell proliferation assay kit (Bet Haemek, Biological Industries, Israel). U87 cells were seeded in 96-well plates (5 × 10^3^ cells/well) and incubated for 24 h. The medium was then removed, the tested molecules (10^−13^-10^−4^ M final concentration) were added in FCS-free medium and introduced to the cells for 72 h. After choosing SIXAC as the leading molecule, it was tested in a similar way in CNS-1 cells (SIXAC 1 μM final concentration). XTT solution (50 μl) was added to each well and incubated for 3 h at 37°C. The optical density (OD) of each well was recorded at 450 and 630 nm in a microplate reader (Infinite M200, Tecan, Durham, NC, USA). Cell viability was expressed as the percentage (ODtest–ODblank)/(ODcontrol–ODblank), where ODtest is the optical density of cells exposed to SIXAC treatment, ODcontrol is the optical density of the control sample, and ODblank is the optical density of wells without glioma cells.

### Western Blot Analysis

Cells were seeded (1 × 10^6^ cells/75 cm^2^ flask) and incubated for 24 h. Then the medium was replaced by FCS-free medium for another 24 h. The cells were treated for 10 min with thrombin (1 U/ml), SIXAC (100 nM), a combination of thrombin and SIXAC or a FCS-free medium which was used as a control (*n* = 3 for each treatment). The cells were washed and lysed in RIPA buffer (containing in mM: 50 TRIS HCl pH 8, 150 NaCl, 1% NP-40, 0.5% sodium deoxycholate, and 0.1% SDS), a protease inhibitor cocktail (Merck Millipore 539134, 1:100), 0.1 mM sodium orthovanadate and 2 mM PMSF. The cells were scraped, collected and centrifuged (16,000g × 20 min) at 4°C. The supernatants were collected, and protein concentration was determined by means of a bicinchoninic acid (BCA) assay. Each sample (20 μg) was separated by SDS-polyacrylamide gel electrophoresis. The proteins were transferred onto nitrocellulose membranes. Membranes were incubated with mouse anti-phosphorylated Extracellular signal-Regulated Kinase (pERK) antibody (1:10,000, M8159, Sigma), for 1.5 h at room temperature (RT) and washed with Tris-buffered saline and 0.1% Tween 20 (TBST). Membranes were then incubated at RT with horseradish peroxidase-conjugated goat anti-mouse antibody (1:10,000, Jackson Immunoresearch Laboratories). Upon detection, the membranes were stripped and re-incubated with a rabbit anti-ERK antibody (1:1,000, sc-154, Santa Cruz Biotechnology). Protein bands were detected by a peroxidase-based enhanced chemiluminescence (ECL) method. Analysis of the protein band density was performed with ImageJ software.

### Wound Healing Assay

The effect of SIXAC on the coordinated movement of the glioma cells was evaluated using a wound healing assay. The cells were seeded in 24-well plates at a density of 5 × 10^4^cells/well. Cells were cultures in FCS-free medium for 24 h. A wound was established by scraping using 10 μl pipette tip. Cells were washed twice with warm PBS and fresh FCS-free medium containing 1 μM SIXAC was added. Image acquisition of wound gap closure (%) was carried out following 4 h of incubation using a Nikon Eclipse microscope. Images were analyzed using Fiji software (37).

### Colony Formation Assay

The cells were seeded in 6-well plates at a density of 100 cells/well. After 24 h, fresh medium with 1 μM SIXAC was added. Cells were incubated for 5 days, until colonies were formed. Cells were washed with PBS, stained with 0.5 M crystal violet (Sigma-Aldrich, C0075) in absolute ethanol for 20 min and then washed again with PBS and dried for 24 h. Colonies that were bigger than 1 mm were counted using Fiji software (37–40).

### Coagulation in Plasma

HemosIL normal control (Instrumentation Laboratory), was used as human normal pooled citrated plasma from healthy donors. The lyophilized human plasma containing buffer, stabilizers and preservatives, was reconstituted using standard laboratory methods. The plasma was exposed *in-vitro* to indicated concentrations of SIXAC for 10 min. Next, prothrombin time (PT), activated partial thromboplastin time (aPTT) and thrombin time (TT) were measured. All tests were carried out with an ACLTOP® 500 autoanalyzerat in the Sheba medical center (Israel) MegaLab according to standard operating procedures (SOP). The results represent an average of 5 measures.

### *In-vivo* Experiments

#### Animals

Adult Lewis rats (270–335 g, Harlan, Jerusalem, Israel) were housed in a standard conditions and fed a standard diet. Water was available *ad libitum*. Ambient temperature was set to 22°C to 23°C with day/night light control. The protocols of this study were approved by the Sheba Medical Center Committee on the Use and Care of Animals (permit No: 972/15) according to the ARRIVE Guidelines. Maximum efforts were made in order to minimize suffering and pain. The humane endpoints were the inability to rise or ambulate and a severe weight loss (20% of initial body weight).

### Tumor Implantation

The rats were anesthetized via IM injection of Ketamine-Xylazine (100 mg/kg, 16 mg/kg, respectively). A midline scalp incision was made, and a 1 mm burr hole was drilled (coordinates 1 mm lateral × 3 mm posterior to the Bregma). Tumor implantation was performed using a 30 gauge needle attached to a 25 μl syringe containing 10 μl of CNS-1 pellet 5 × 10^7^cells/ml. The rats were positioned into a stereotactic frame in order to inject the cells at the right corpus striatum 5.5 mm deep in the brain tissue. Cells were injected with a syringe pump at a rate of 2 μl/min for 5 min; hence the final number of implanted cells was 5 × 10^5^. The needle was gently pulled out 1 min after the pump was stopped. The burr hole was then sealed with bone wax and the skin was sutured.

### MRI Data Acquisition

MRI was performed using a clinical interventional 1.5T MR system. The rats were anesthetized and Gd-based contrast agent was injected IP (Gd-DOTA, 0.02 mmol/kg, Dotarem, Guerbert). MRI sequences included contrast enhanced T1-weighted MRI for depiction of the tumor, T2-weighted MRI for depiction of edema and gradient-echo MRI for depiction of possible hemorrhages. The first MRI scan was used to rank the tumors into three categories based on the size evaluation in order to form groups with a similar distribution of initial tumor size. Regions of interest (ROIs) were plotted over enhanced T1 and T2 weighted MR images. The volume of the tumors, calculated from the T1-weighted images, was then subtracted from the enhancing volume, calculated from the T2-weighted images, in order to determine the volume of surrounding edema.

### Alzet Osmotic Pump Implantation

The animals were implanted with osmotic pumps attached to brain infusion tube (Alzet, Cupertino, CA, USA). Implantation was performed 5 days after tumor inoculation. Two different types of osmotic pump were used: in the imaging study the pumps implanted released 1 μl/h for 7 days (model #2001), while in the survival study the pumps released 0.5 μl/h for 14 days (model #2002). The pumps were preloaded with either saline or SIXAC (in indicated concentrations). The pumps were primed in saline bath at 37°C for 24 h as a pre-implantation procedure and the function of each pump was reassured by assessing dripping. The Alzet pump was implanted subcutaneously, a catheter was placed intracranially thus releasing substances directly to the tumor core.

### Experimental Design

The effects of SIXAC on tumor and edema volume and on survival were studied in two separate experiments. Repeated MRI scanning for tumor and edema quantification requires multiple anesthetic procedures that are known to increase mortality rate, and thus might bias the survival study.

#### Imaging Study

Rats were allocated to three groups. 1. Control group (*n* = 13) treated with alzet-pump containing saline. 2. Low dose treated group (*n* = 14) treated with alzet-pump containing SIXAC (0.2_μ*g*/*kg*/*day*_). 3. Moderate dose treated group (*n* = 14) treated with alzet-pump containing 10 times higher SIXAC concentration (2_μ*g*/*kg*/*day*_). 7 days following induction of treatment (day 12) a follow-up MRI imaging (2nd) was performed and the size of the tumor and edema surrounding the tumor were analyzed by standard ROI manual techniques as described above. In order to eliminate the effect of initial tumor and edema sizes variance, the tumor and edema sizes increase were calculated by using the ratio of the 2nd MRI scan divided by the 1st MRI scan (41, 42).

#### Survival Study

Rats were allocated to three groups. 1. Control group (*n* = 15) treated with alzet-pumps contain saline. 2. Moderate dose treated group (*n* = 16) treated with alzet-pump containing SIXAC (2_μ*g*/*kg*/*day*_). 3. High dose treated group (*n* = 14) treated with alzet-pumps contain SIXAC (20_μ*g*/*kg*/*day*_). Survival was registered daily and the brains were dissected immediately after death determination and were placed in 4% PFA and kept at 4°C. Upon MRI scanning the brains were moved to PBS containing tubes, scanning was performed and ROI were quantified.

### Pathological Evaluation of the Tumors

Formalin-fixed, paraffin-embedded brains were serially sectioned into 4 μm sections and stained with Hematoxylin and Eosin (H&E). Tumor localization and detailed evaluation was conducted by a senior neuropathologist (NS). Mitosis rate was assessed manually by 2 observers who analyzed 10 fields from each examined brain. Serial 4 μm sections were used for immune-histochemical staining using a commercially available monoclonal rabbit anti-Ki67 antibody (clone SP6,# RM-9106-S, Thermo Scientific, 1:300) according to standard procedures and fully automated protocol. A positive control was added on the right side of the slides for immune-histochemistry. The slides were automatically scanned via Aperio Versa 200 System (leica) using X20 and X40 equivalent objectives. Digital images were captured with Leica DM6000B microscope by AperioImageScope software. The Ki67 index was defined as positive stained nuclei (DAB positive)/all stained nuclei. Ten fields from each tested brain were used for analysis of Ki67 index. The indices were automatically calculated by means of ImmunoRatio application (Version 1.0c) (43).

### Statistical Analysis

Results are expressed as mean ± standard error of the mean (SEM). Statistical analysis were carried out using a One-way ANOVA followed by Dunnet's *post-hoc* analysis, *t*-tests and log-rank (Mental-Cox) test. Cutoff for statistical significance was set at *p* < 0.05. The analyses were performed using SPSS software (Chicago, Illinois), GraphPad Prism software (San Diego, California, version 6.01) and by using Excel statistical functions (Microsoft Corporation).

## Results

### PAR1 (F2R) Is Overexpressed in Human Brain Glioma Patients

We conducted a differential analysis for F2R gene expression in human GBM and control brain by using an Oncomine dataset based on the TCGA database. The F2R overexpression gene rank was found to be 236 which means it is within the top 2% of genes that are overexpressed in GBM patients. The F2R expression levels were significantly higher in the GBM tissues compared to normal brain tissue (fold change 16.46, t = 28.9, *p* = 9.38E-12) (Figure 1A).

### Characterization of Molecules

C6 cells have been shown to produce and secrete thrombin (19). We measured the ability of the molecules to inhibit thrombin activity secreted from C6 as a screening test in order to characterize the leading molecule. The measured activity was inhibited by all the tested molecules in a dose dependent manner (Figure 1C). Two of the molecules, 6AACK and 7AACK, were found to be the most potent in inhibiting thrombin activity (calculated IC50 of 2.6 × 10^−14^M and 1.2 × 10^−14^M, respectively). The 3AACK and 4AACK molecules were found to have the highest calculated IC50 (3.4 × 10^−10^ and 1.3 × 10^−9^M, respectively) and were therefore less potent.

We further studied the proliferation inhibiting potential of the molecules in U87 human glioma cell culture. Although it appears that 3AACK inhibits more efficiently the proliferation rate when compared to 6AACK at a concentration of 10^−8^M, this was found to be non-significant (Figure 1D, *p* = 0.7). Interestingly, the 6AACK molecule was found to inhibit the U87 proliferation rate the most (Figure 1D, 3AACK and 6ACCK at 10^−5^M found to inhibit proliferation at a rate of 0.58 ± 0.046, 0.2 ± 0.34, respectively, *p* < 0.035).

Taking the thrombin inhibition and proliferation test results together, 6AACK was found to have the highest potency to inhibit both glioma proliferation and secreted thrombin activity *in-vitro*. Thus, 6AACK renamed SIXAC was chosen as a leading molecule for further study.

### *In-vitro* SIXAC Characterization

**Figure 2:**
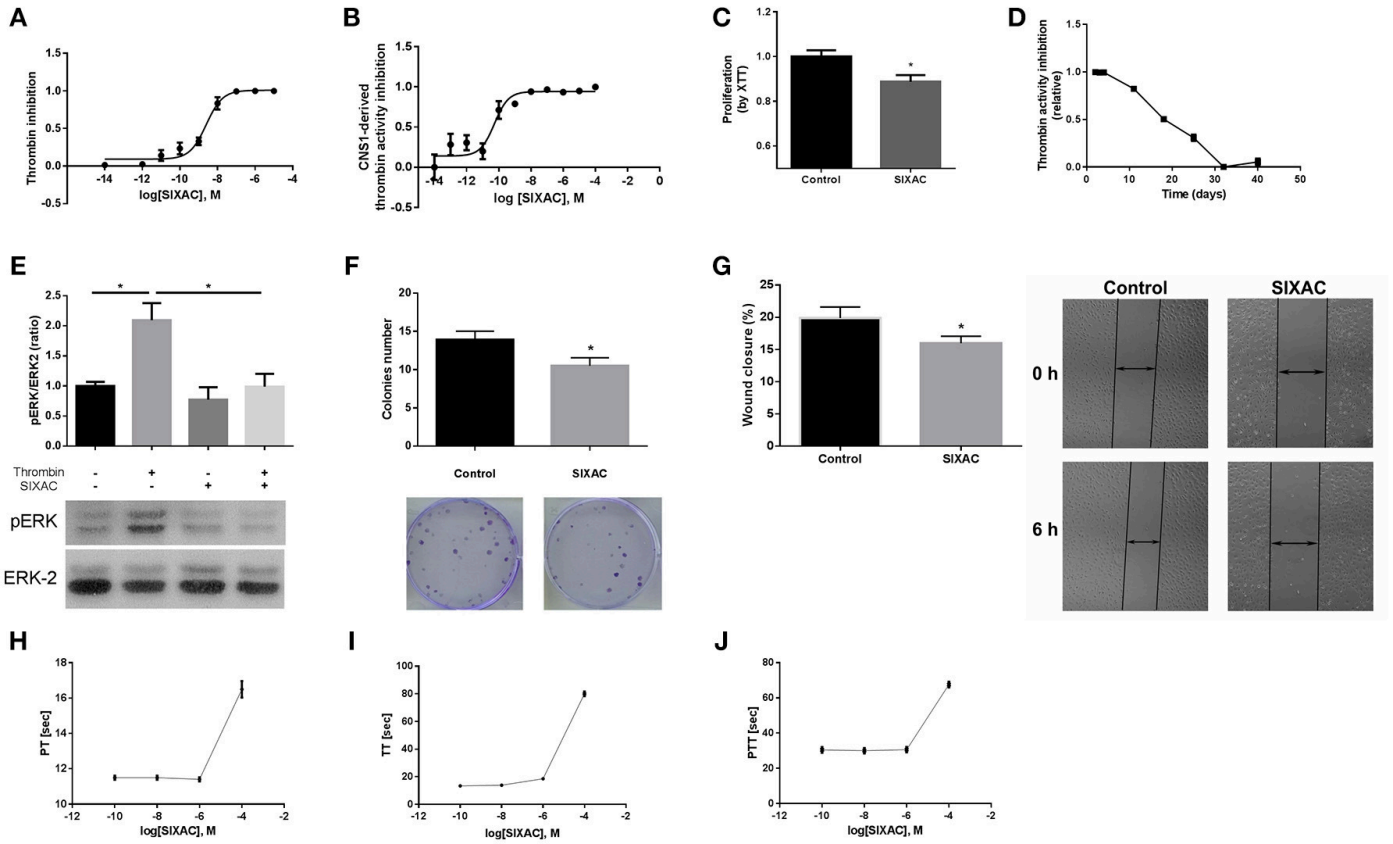
SIXAC *in-vitro* effects on glioma cells. **(A)** Inhibition of commercial thrombin activity in a concentration dependent manner. **(B)** Inhibition of thrombin activity derived from CNS-1 glioma cells in a concentration dependent manner. **(C)** Inhibition of CNS-1 glioma cells proliferation rate deteceted by means of the XTT assay. **(D)** SIXAC stability over time at 37°C measured by its inhibition of thrombin. **(E)** Inhibition of thrombin mediated PAR1 activition by SIXAC as measured by activation of ERK (pERK/ERK ratio). **(F)** Inhibitory effect of SIXAC on the CNS-1 colonies formed **(G)** Inhibitory effect of SIXAC on wound closure percentage. **(H–J)** Direct effect of increasing concentrations of SIXAC on the indicated blood coagulation tests. All assays were performed as described in the methods section.

In order to characterize the ability of the SIXAC molecule to specifically inhibit thrombin activity, we measured the inhibitory effect of this compound on commercial thrombin as described in the methods. As can be seen in Figure 2A, SIXAC inhibited measured thrombin activity in a concentration dependent manner. We calculated the IC_50_ for 0.05 U/ml commercial thrombin to be 2.5 × 10^−9^M SIXAC. As known, glioma cell lines generate thrombin activity that can be detected in the cell culture medium (5). We therefore further tested the ability of SIXAC to specifically inhibit the glioma-derived thrombin activity. As can be seen in Figure 2B, increased SIXAC caused an inhibition of the thrombin activity measured in the surrounding medium of the CNS-1 cell line. This effect was found to be concentration dependent and the IC_50_ was calculated to be 5 × 10^−11^M SIXAC. The glioma-secreted thrombin activity, which is known to be essential for cell survival and proliferation, may hold a role in tumor proliferation and edema generation. Therefore, we studied the SIXAC effect on proliferation rate of the CNS-1 cell culture. SIXAC was found to significantly decrease the CNS-1 cell proliferation rate (Figure 2C).

In order to confirm that SIXAC inhibits thrombin activation of PAR1, we measured activation of ERK by phosphorylation (pERK), the major pathway activated by PAR1 in CNS-1 cells. As can be seen in Figure 2E, pERK/ERK ratio was increased significantly by thrombin (2.09 ± 0.28 vs. 1 ± 0.07, *p* < 0.03), indicating that thrombin activated PAR1. The increase of pERK/ERK ratio was prevented in CNS-1 cells exposed to thrombin with SIXAC compared to thrombin alone (2.09 ± 0.28 vs 0.99 ± 0.21, *p* < 0.03). In order to expand the understanding of the cellular mechanisims affected by SIXAC, we conducted two more sets of *in-vitro* experiments. A colony formation assay was conducted and found SIXAC significantly reduced cell proliferation indicated by the lower number of formed colonies (Figure 2F, 13.9 ± 1.09 vs. 10.5 ± 1.06, *p* = 0.033). A second set of experiments was conducted by means of a wound-closure assay. SIXAC was found to significantly decrease the wound-closure percentage (Figure 2G, 10.14 ± 0.67% vs. 7.27 ± 0.68%, respectively, *p* = 0.02). The significant effect on wound-closure percentage indicates SIXAC reduces cell-migration and invasiveness *in-vitro*.

Since SIXAC was found to inhibit both commercial and cell-derived thrombin activity *in-vitro*, it was important to characterizing SIXAC therapeutic window and define the threshold above it coagulation side-effects might occur. We conducted PT, aPTT, and TT tests and found no clinical significant effects for SIXAC at concentrations equivalent to those used to treat animals (Figures 2H–J). SIXAC was found to affect coagulation tests only at 10^−4^M which is at least 4.5 orders of magnitude higher than its IC_50_ for thrombin inhibition. This strongly supports SIXAC to be safe in terms of bleeding, even in systemic administration. SIXAC was found to be stable and significantly inhibit thrombin in physiological resembling conditions for as long as 18 days at 37°C (Figure 2D). This data indicates that SIXAC administered in an Alzet osmotic pump for 14 days would retain its ability to inhibit thrombin.

### Imaging Study

**Figure 3:**
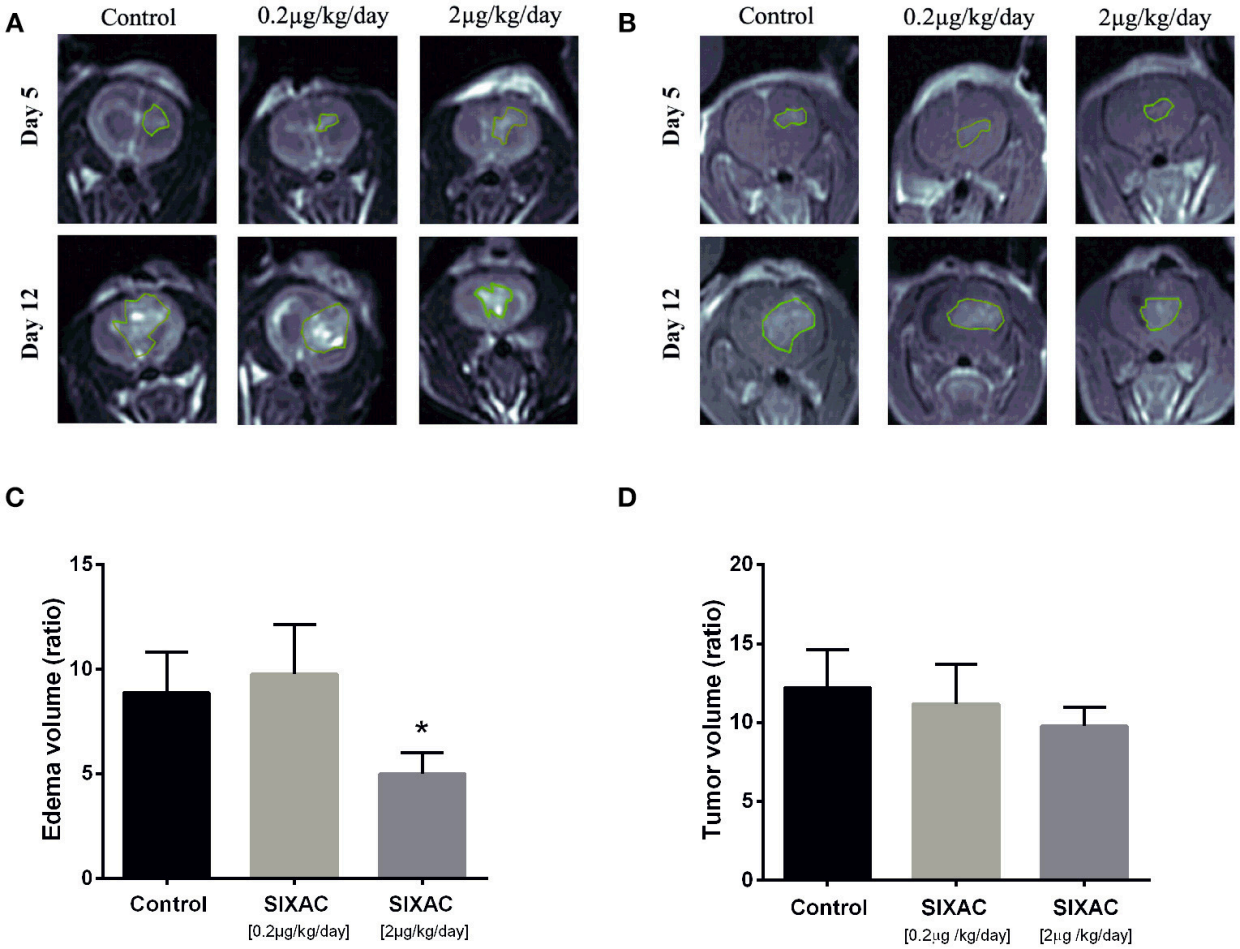
SIXAC treatment effect on edema and tumor volume ratios in a rat GBM model. **(A)** Representative coronal view of T2-weighted MR images, hyper-intense regions comprised of tumor tissue with its surrounding edema in saline, low dose and moderate dose SIXAC treated rat groups. **(B)** Representative coronal view of T1-weighted MR images, hyper-intense regions comprised of tumor tissue in saline, low dose and moderate dose SIXAC treated rat groups. **(C)** Tumor edema ratio plots were calculated as described in the methods section from data measured by MRI performed on day 5 after tumor inoculation and on day 12 following 1 week of treatment. Significant reduction in edema means of ratios was found when comparing the moderate dose group (2_μ*g*/*kg*/*day*_) to low dose (0.2_μ*g*/*kg*/*day*_) and saline groups. **(D)** Tumor enhancement ratio plot was calculated as described in the methods from data measured by MRI performed on day 5 after tumor inoculation and on day 12 following 1 week of treatment. A non-significant reduction in tumor enhancement means of ratios was found when comparing moderate dose group (2_μ*g*/*kg*/*day*_) to low dose (0.2_μ*g*/*kg*/*day*_) and saline. *p < 0.05.

The inhibition of thrombin activity and CNS-1 cell proliferation by SIXAC *in-vitro* led us to examine its effects *in-vivo*. We examined the effect of SIXAC on tumor and edema size by comparing the MRI scans between three study groups as described in the experimental design section of the Methods. Out of 41 CNS-1 inoculated rats, only 20 survived to undergo the 2nd MRI scan (day 12) including 6 rats in the control group, 5 rats in the low dose group (0.2_μ*g*/*kg*/*day*_) and 9 in the moderate dose treated group (2_μ*g*/*kg*/*day*_). Edema (T2 hyperintense) and tumor (enhancing lesions) ratios of each group (representative images can be seen in Figures 3A,B) were quantified and plotted in Figures 3C,D, respectively. In the *in-vivo* imaging study we administered 2 concentrations of SIXAC which we defined as low and moderate doses. The more robust and easily measurable index tumor size was the T2 hyper intensity which essentially measures both tumor and edema. Using this measure of tumor size, a significant lower edema volume ratio was found in the moderate SIXAC concentration (2_μ*g*/*kg*/*day*_) compared to control (4.9 ± 1 vs. 8.8 ± 1.9, *t*-test. *p* < 0.04). When the smaller and more variable size of enhancing lesions were assessed as measures of tumor size, a non-significant decrease in tumor volume ratio was found when comparing moderate concentration (2_μ*g*/*kg*/*day*_) to control (9.8 ± 1.2 vs. 12.2 ± 2.4, *t*-test, *p* = 0.16).

### Survival Study and Histopathology

**Figure 4:**
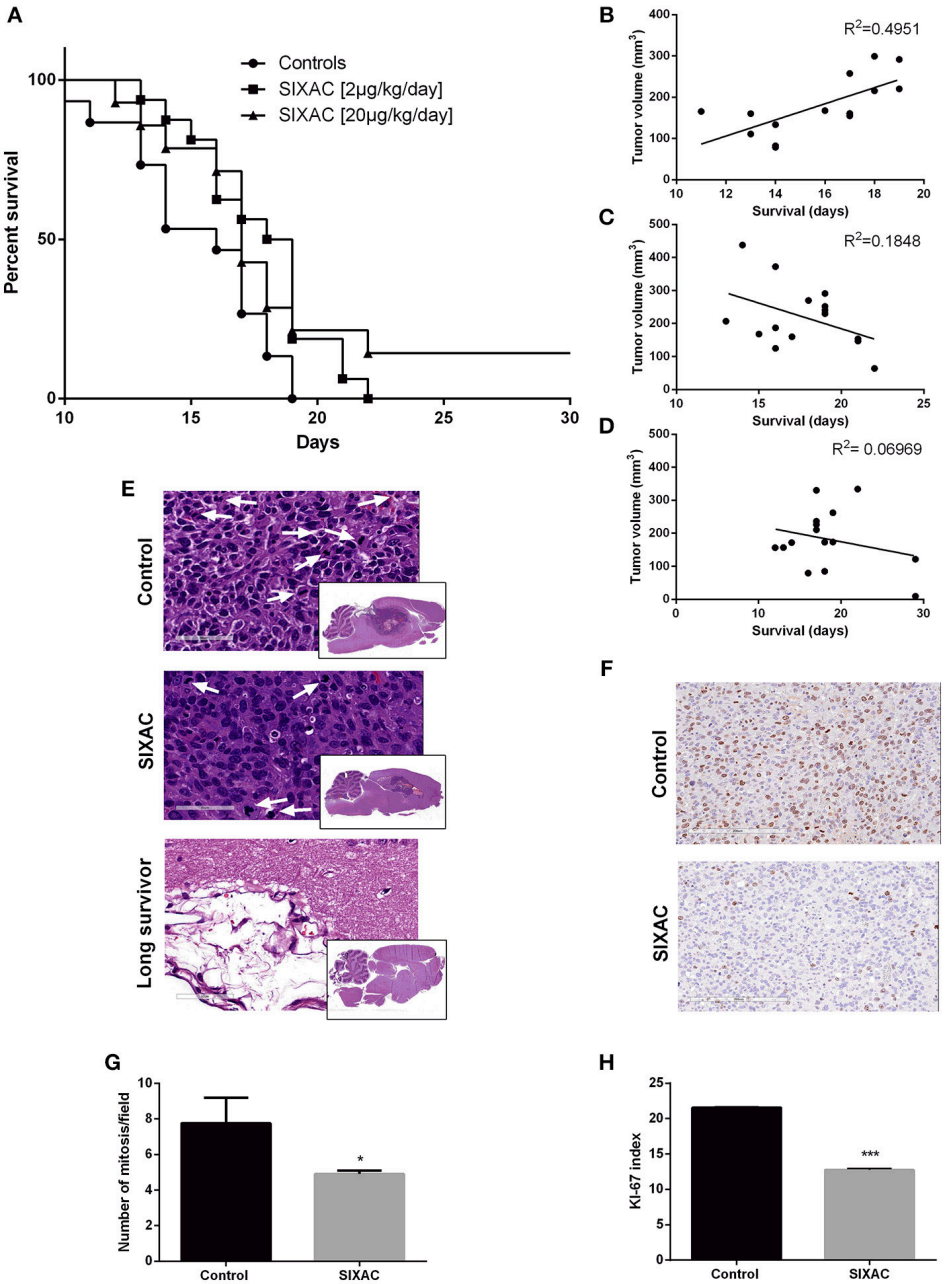
Improved survival and histopathological measures in GBM CNS-1 model rats treated by SIXAC. **(A)** Kaplan-Meier curves comparing saline treated rats (circles) to groups of high (triangles) and moderate (squares) dose of SIXAC treated rats. There was a significant trend of prolonged survival in comparing the three groups (log-rank test for trend; *p* = 0.030). The effect of the moderate dose treatment on survival was more pronounced (log-rank test; *p* = 0.01) than the effect of the low dose (*p* < 0.05) in comparison to the saline treated group. PM brain tumor volumes in correlation to survival in days. Linear regression lines and Pearson *R*^2^ values are shown for each group. **(B)** Control group, **(C)** Moderate dose treated group, **(D)** High dose treated group. Number of animals: control = 15, SIXAC moderate dose = 16, SIXAC high dose = 14. **(E)** Representative H&E brain stainings of control, SIXAC, and “long survivors” groups (magnification 10X). White arrows indicate mitosis (magnification 400X). **(F)** Representative Ki67 immunostaining of control and SIXAC group. **(G)** Number of mitosis/field as was calculated from H&E staining. **(H)** Ki67 index as was calculated using the ImmunoRatio application. Results are presented as mean ± SEM. *p < 0.05, ***p < 0.001.

We compared the survival rate of CNS-1 inoculated rats treated intracranially with either saline, moderate dose (2_μ*g*/*kg*/*day*_) or high dose (20_μ*g*/*kg*/*day*_) SIXAC. 43 rats died during the first 17 days post tumor inoculation. 2 rats from the high dose SIXAC group were long survivors despite evidence of tumor inoculation by MRI. These rats were sacrificed at day 40. The median survival was 16 days in the control group vs. 18.5 and 17 days in the 2_μ*g*/*kg*/*day*_ and 20_μ*g*/*kg*/*day*_ SIXAC groups, respectively. We found a significant trend of prolonged survival when comparing the three groups (log-rank test for trend; *p* = 0.030) as can be seen in Figure 4A, which depicts Kaplan-Meier curves of the three different treatment groups. Close inspection of the survival curves in Figure 4A reveals high similarity between the moderate and high SIXAC doses. For an early brief time period, survival in the higher SIXAC dose was lower. In the late phase, survival was higher in the high dose group, due to 2 unusually long-term survivors. The SIXAC treatment groups are therefore very similar and combining these groups relative to controls reveals a robust effect of treatment. When comparing the individual SIXAC treatment groups to controls, we found that the survival rate of the moderate dose group (2_μ*g*/*kg*/*day*_) was significantly prolonged in comparison to the saline treated group (log-rank test; one tailed *p* = 0.01) with a less pronounced effect for the high dose group (log-rank test; one tailed *p* < 0.05).

A linear regression model which correlates PM brain tumor volume with survival day in each group is presented in Figures 4B–D. The results indicate moderate positive correlation between the final tumor size and survival duration in the control group (y = 19.56x-129, *R*^2^ = 0.49 by Goodness of Fit test, *p* = 0.005), compatible with the linear growth of the tumor in these animals. Interestingly, the *R*^2^ was not significant in the treatment groups (moderate dose 2_μ*g*/*kg*/*day*_, *p* = 0.1, high dose 20_μ*g*/*kg*/*day*_, *p* = 0.3417) and the regression lines are compatible with a negative relationship between final tumor volume and survival duration which may indicate an inhibition of the tumor growth pattern observed in the control group (2_μ*g*/*kg*/*day*_: y = −15.57x+495.4, 20_μ*g*/*kg*/*day*:_ y = −4.78x+270). In order to further study the effects of SIXAC on tumor cellular processes in the brain at a microscopic level we conducted H&E and immunostaining.

Representative H&E slides are presented in Figure 4E and demonstrate the existence of large tumor and its known characteristics as high cellularity, pseudopallisading, necrotic centers, atypical mitosis, apoptotic cells, intravascular thrombosis and ischemic necrosis. A representative H&E stained-slide from a long survivor rat demonstrates a large cystic area containing residual tissue with foamy macrophages compatible with an inflammatory reaction to tissue destruction. The localization of this cystic area is highly analogous to the area under the corpus callosum occupied by tumor in the control rats. The mitosis rate measured in H&E stained slides was found to be significantly lower in the SIXAC treated rats when comparing to the control (4.9 ± 0.2 vs. 7.75 ± 1.45, *p* = 0.03, Figures 4E,G). Rats treated with SIXAC showed a reduced Ki67 index compared to control GBM rats (12.74 ± 0.23 vs. 21.55 ± 0.11, *p* < 0.001, Figures 4F,H). These results support an effect of SIXAC on tumor proliferation and infiltration.

## Discussion

A growing body of evidence from the last decade indicates the fundamental involvement of brain thrombin and PAR1 in the progression of glioma (1, 3, 4, 10, 11). The TCGA added a vast network of information regarding genes of interest in glioblastomas patients, and possible research directions (44). Indeed, by using datasets from the TCGA database we found that PAR1 is one of the top 2% of genes being overexpressed among GBM patients. This finding strongly suggests PAR1 as a target for intervention in the treatment of GBM. In accordance with previous studies, the CNS-1 tumor cell line expresses PAR1, implicating the receptor as a target for auto-activation by thrombin or thrombin-like proteases (membrane tethered or secreted by the tumor *in-vivo*). Thrombin affects neuro-glial transmission, cellular signaling, inflammation, differentiation, pathological proliferation, malignant invasion of glial cells, and tumor associated brain edema (15, 19). We developed and synthesized a series of compounds that inhibit the specific proteolytic activation of PAR1, at the thrombin cleavage site. These novel compounds were modified by addition of tosyl in order to prevent their degradation by amino peptidases, and by chloromethylketone (CMK) which irreversibly inhibits thrombin-like proteases, thus increasing their stability and potency. The molecules were characterized in their ability to inhibit glioma cell proliferation and secreted thrombin inhibition. Based on the characterization results a six amino acid backboned molecule was chosen as the leading compound (named SIXAC). Altough SIXAC is relatively specific to the thrombin recognition site sequence on PAR1, it potentially targets other proteases that hold a common recognition site such as FXa (45). As described above, we conducted a series of experiments in order to evaluate the effect of SIXAC on coagulation measurement. The effect was minimal for the therapeutic dosages used in the experiment.

SIXAC inhibited CNS-1 proliferation and migration in a mechanism directly related to the MAPK pathway. Inhibitory effects were measured in both colony formation and wound closure assays. SIXAC influence on PAR1 was indirectly measured via the pERK/ERK2 ratio. PAR1 activation is known to induce proliferation in astrocytes via MAPK involving multiple signaling pathways (46).

In this study we present for the first time the *in-vivo* effects of SIXAC on the CNS-1 cell glioma in tumor implanted rodents.

In the ***in-vivo* imaging study** we found a significant reduction of edema mean ratios when comparing moderate dose (2_μ*g*/*kg*/*day*_) and saline treated groups. A non-significant trend toward smaller tumor volume ratios was found for the SIXAC treatment. This might be explained by the limited number of rats that survived to undergo follow up MRI (saline *n* = 6, low-dose *n* = 5, moderate-dose *n* = 9) probably due to high mortality rate following repeated anesthesia procedures.

**The survival study** found that treating CNS-1 glioma inoculated rats with SIXAC prolongs survival. In addition, PM tumor volume in relation to survival days indicated that SIXAC significantly inhibited tumor growth. In the controls a significant linear growth in tumor volume was found with time but in the treatment groups this trend was reversed. These results suggest an effect of SIXAC reducing tumor growth, edema and eventually increasing survival.

The CNS-1 cell line used in the present study is known to recapitulate many of the key characteristics of GBM growth pattern, invasive nature, angiogenesis and microenvironment (25). These GBM characteristics makes this a “double-edged sword” model. On the one hand achieving beneficial results using this model will probably lead to a more pronounced effect in human GBM than other models. On the other hand it is known to be an aggressive model, leading to faster tumor growth and enhanced invasiveness (25); thus it is extremely difficult to detect a beneficial effect for a tested drug. It is only reasonable to infer that using this model strengthens the significant results aforementioned while obscuring some of the beneficial effects. In comparison with the C6 model, the CNS-1 model triggers a weaker immune response, and therefore less rejection. This trait makes the CNS-1 model closer to the human disease, and therefore, better suited for this study (25).

Two rats of the high dose group in the **survival effect study**, survived until sacrificed at day 40. Though both rats were tumor inoculated and definite tumors were found both by MRI at treatment initiation and by PM inspection of their brains, they survived 23 more days than the median survival which was 17 (all three groups). In both studies, PM examination was done on all rats, removing the Alzet pump, opening the cranium and removing the brain. This procedure on these two rats revealed macroscopically that the tip of the Alzet pump was askew and not directed into the tumor itself but still in the cranial compartment. When dealing with a future oncologic directed treatment, one should sometimes inspect the extreme responders. Future research is needed in order to study the optimal SIXAC administration, other PAR1 inhibitors and perhaps less aggressive animal models.

In conclusion, we treated CNS-1 glioma injected rats with SIXAC via an implantable Alzet pump and characterized the *in-vivo* effects regarding tumor growth, surrounding edema, and survival. The positive results we have found in this study demonstrate that PAR1 pathway modulation holds beneficial effects on glioma progression and strengthen this strategy. Our results suggest that SIXAC will be a very interesting compound in both studying and modulating PAR1 in GBM. We hope that future treatment with SIXAC can be an add-on therapy to the accepted radiochemotherpy and improve survival in GBM patients, as well as potentially their quality of life by reducing tumor-associated edema.

## Author Contributions

JC as Chief Scientist and ES-S devised the project, the main conceptual ideas and proof outline. ES-S and ES worked out most of the technical details and performed the *in-vitro* and *in vivo* experiments. NM and AD contributed their knowledge and scientific expertise in theoretical background and study design. SH, ZZ brought their neurosurgical knowledge in scientific background and de facto with the help of AW and ES. VG, OG, KF, and ZI-H were major contributors to all studies from designing of molecules to giving a hand in *in-vivo* studies. SS, DL, DG, and DD of the advance technology center constructed the imaging study. Moreover, under the guidance of YM helped in analyzing imaging data. NS as neuropathologists, advised regarding histopathology and operated staining of slides. SG conducted editing and rewriting of the manuscript, statistical analysis and figures editing. IT gave his productive input regarding cellular models and studies. NR advised and conducted the characterization of SIXAC molecule.

### Conflict of Interest Statement

The authors declare that the research was conducted in the absence of any commercial or financial relationships that could be construed as a potential conflict of interest. The reviewer KC and handling Editor declared their shared affiliation.
